# Etiology and Pathology of Dental Caries and Treatment of Pulps

**Published:** 1883-06

**Authors:** J. R. Porre


THE DENTAL REGISTER.

VOL. XXXVII.] JUNE, 1883. [No. 6.

### COMMUNICATIONS.

### Etiology and Pathology of Dental Caries and Treatment of Pulps.

BY DR. J. R. PORRE.

[Read before the Miss. Valley Dental Society.]

MR. PRESIDENT.—The etiology of dental caries has long been under investigation and controversy by the ablest observers in our profession. And to-day we stand where we did forty years ago, so far as any fixed or accepted principle is concerned. Theories are numerous, many of them are more or less plausible. The latest promulgated, discussed, and backed by considerable ability and much scientific research is the fungi theory. The agency of low organisms, bacteria, bacilli, micrococci, leptothrix, etc., etc.

That they decalcify and penetrate the enamel through the canaliculi, by a process of impaction and fermentation, destroying the dentinal fibrils, etc., is advocated by some. By others, that decalcification occurs first by the action of acids generated or received in the mouth, followed by the invasion of the organisms, etc., etc. Lime salts are either materially wanting in the original structure of the teeth or they are extracted by some agency, before caries and the putrefactive process begin. The acid theory, from whatever cause or combination of causes, specific or general, is a rational one, according to the laws of cause and effect, and in the light of chemical influences and action. I shall defer the discussion of this subject to others and occupy your attention with phases of the question that I regard of great practical importance, which I desire to present for the consideration of this meeting. You will, therefore, pardon me for the digression. The principle, I believe, is taught and generally practiced by the profession, to remove as little of the dentine over the pulp in large cavities preparatory to filling as is at all compatible with its protection from pressure. Excavating thoroughly around the margins and walls, but leaving a covering of dentine, however disorganized, broken, or porous it may be, depending on its saturation with carbolic acid or creosote, etc., to impart to it a preservative and non-irritative quality. Treating the pulp meanwhile with the aim to place it in a normal condition and to insure immunity from disturbance thereafter. The design of this paper is to take issue with this practice as unphilosophical and impracticable, utterly opposed to every principle of therapeutics as taught and practiced in application to pathological conditions prevailing either in hard or soft tissues in every other part of the human organism. If caries or necrosis involves the alveolus or maxillaries or other hard tissues of the body, an operation for their removal is absolutely required before a cure can be effected. So of ulceration and gangrene which bear the same relation to the soft as caries and necrosis do to the hard tissues.

The importance of this subject prompts me to be thus plain and to the point. Now to the question: Is it correct practice to leave dead, porous dentine as a covering or capping over pulps preparatory to filling? Emphatically no. I repeat, it is antagonistic to every principle of physiology and the consistent laws of hygiene and therapeutics. Local lesions, traumatic or pathological, are met by an effort of nature with her own peculiar processes to effect repair. When the soft-tissue covered bones are injured or fractured, after being properly dressed they are restored to former usefulness, leaving scarcely a trace of the lesion discoverable. Not so with the teeth, they being the only exposed bones of the body are consequently subject to opposing and irritating agencies that preclude reconstruction or restoration from either caries or fracture. Yet the same phenomena attend lesions as with other bones. Inflammatory action with effusion of lymph, in a word, all the materials and aids, are generally furnished notwithstanding the failure or impossibility to be utilized or appropriated. Nature but makes the ineffectual effort. This process is exhibited often, even when the organ is involved to a limited extent with caries, to affect the pulp through the tubuli with inflammatory action. At first the disturbance is but slight, perhaps, but as the disorganization extends and with the action of extraneous putrefactive and irritating causes, the pulp becomes inflamed, then with the increased vascularity and consequent engorgment and for reason of a lack of accomodating space, odontalgia is the result. It is a well known fact that the products of inflammatory action in their chemical characteristics contain a large excess of the mineral salts, chloride of sodium, and the phosphates; *i. e.*, pus, lymph and the serum contain three times as much as normally. So the effusion of lymph laden with these elements, excited by a dental nerve invaded by caries, indicates a purpose of nature which we should not disregard. It is a fact, as stated, that no reconstruction of bone or deposit of secondary dentine can take place in a cavity lined with dead tissue. Its contact with the pulp is immediately or will be remotely, an irritant to excite inflammatory action in a large number of cases, however carefully it may be treated. Being a dead, decomposing, disintegrating section of animal tissue, it is a foreign body, from which nature recoils with disgust and will resent sooner or later, with distressing local and general disturbance. This "leathery" permeable dentine, therefore, should be thoroughly extirpated, even if necessary to the exposure of the pulp, not only for the reasons that have been given but for another most important one. A diagnosis cannot be correct as to the condition of a nerve concealed or covered, as above described. By treatment a deranged nerve may be quieted and kept under surveillance for a satisfactory period, perhaps, as a test; but after permanently filling, in time, the antiseptic is absorbed, then upon some general disturbance of the system supervening, the pulp manifests its imperfect protection and treatment in pronounced rebellion. The peculiar restraining influence of the medicant being gone nature resumes her processes again for repair. Of course much depends upon the stage or extent of disorganization of the dentine, the age and standard of health and circumstances connected with the patient. When the nerve is exposed a correct diagnosis can be made in almost every instance. If it is congested or inflamed, its color and the pulsations will show If there is an exudation of sanious fluid or bloody lymph or an excessive effusion of lymph as is very often the case, or if in a state of health, the condition is apparent whatever it may be, and the treatment is indicated accordingly. Then you can treat the case judiciously and understandingly. Now as to treatment of both dentine and nerves. I read a paper before the Cincinnati Dental Society some months since, which was published in one of the dental journals, on the treatment of sensitive dentine, which would equally apply to the conditions under consideration. I will give a synopsis sufficient to serve our purpose: Invest the tooth with rubber dam; have prepared in small glass or porcelain jar one part glycerine and three parts pure carbolic acid and place over a spirit lamp, heat and keep hot but not boiling; then dip with pliers a piece of spunk of size to be easily introduced into the cavity, into the preparation, touch on blotting paper to absorb excess and apply. First few pieces should be barely warm; by gradation the heat can be increased, if necessary at intervals of a few moments, until, by test, the conditions are favorable to proceed to excavate. Repeat the treatment as may be required and always dry the cavity before using the instrument as the temperature and dryness are factors not to be disregarded. As a rule large cavities are less sensitive than small ones, so the difficulties are ordinarily light in preparing them, until the immediate region of the pulp is approached, when the treatment becomes essentially important, not only as an obtundant, but also for therapeutical action, as, taking it for granted, that all nerves under the conditions described are more or less affected, and, too, as a prophylactic. As to the reason why a nerve the most delicate tissue of the human organism, tolerates so active and powerful an escharotic as carbolic acid is a question yet to be satisfactorily explained. That it does and that it is the leading curative, antiseptic, and preservative agent used and relied upon by the profession for the treatment of dental nerves there is no question. But the manner of using it as we recommend may be a subject of surprise and perhaps of decided skepticism as to the effects claimed. But, to proceed, when nearing the nerve, the sensibility is regulated by increasing the temperature, but to be very careful to introduce gradually to avoid the infliction of pain. When every particle of devitalized dentine is removed, if the nerve pains, moisten the spunk in a preparation of equal parts of chloral, gum camphor, and eucalyptus, and apply. If this fails, which it seldom does, try chloroform liniment or any favorite preparation. The manipulation being governed by judgment, and after a few moments' patience, the pain will subside, then if the indications are favorable, cap and fill at once. If they are not, if there is an exudation or an effusion of lymph, moisten a proper sized piece of spunk in the chloral medicant and dip in the warm acid and apply over the nerve and seal with warmed wax and postpone for a day or two. Never allow the saliva to come in contact with the nerve while under treatment—it contains elements antagonistic to success—if not micro-organisms, and we cannot gainsay their presence, then other principles of vitiation and disturbance. I have treated many scores of pulps by this process and have not been compelled to remove the fillings for treatment of more than two or three per cent. Have treated and capped as many as four nerves in one mouth at a sitting, and in that mouth were six capped nerves—two capped a few days previous, which, the patient called to have extracted, because of their infliction of suffering—now over a year since, and the patient, seen a few days since, reported absolute comfort since the day of the operation. In the mouth of another patient are five capped nerves, treated about the same time, with same result. Patients have called to have aching teeth extracted, I have pursuaded some of them to let me treat and fill at once as described; have never had one to return, although urgently requested to do so, if necessary, to report trouble afterwards. If a pulp dies, the tooth shows off color and is easily recognized. Have made examination when opportunity offered and not more than one per cent have devitalized. This result, of course, is attributable to especial care in diagnosis. If a nerve is disorganized the treatment will at once make it manifest by escharotic action and consequently turning the tissue white and after a few applications depriving it of sensibility. In such case, increase the heat of treatment so as to make the operation as painless as may be in the extirpation of the diseased section, and then treat and save the remainder. Carbolic acid will not destroy dental nerve tissue when the structure is not disorganized and disintegrating if applied as treatment according to the simple rules of common sense. If our treatment is applied superheated, of course it will scald the nerve to death. The acid applied ordinarily to any other nerve or soft tissue actively destroys.

But a word on that point later. The arsenious acid bottle is in my case, a relic of the past. Our treatment has entirely superseded it in all cases where the destruction of the nerve, or portions of it, is required. Having the nerves liberated from their dead covering and exposed to view a failure need seldom be recorded. When there is doubt, delay, place the nerve under treatment, not exceeding three days, for the sooner the case is permanently disposed of after favorable indications the better. I first use a paste of one part of spirits of camphor and five parts of carbolic acid tar (which you manufacture in your heating jar, after the acid becomes somewhat consistent, after use by evaporation, then boil down to a tarry consistence) and precipitated creta prep. q. s. Now the manipulation is a very important part of the operation. Deposit of the paste a quantity proportioned to size of nerve exposure, to be covered if no pain, then cover with oxy-phosphate or oxy-chloride, using a small amount and very thin. After it has set add to desired thickness. There must be no pressure or impingment to the least degree. Whether there will be or not a deposit of secondary dentine, cases are reported, there is nothing in the way of an exchange of place with the paste. If not, the paste is not offensive to the nerve. A valuable feature of this process of treatment is the dispatch with which cases can be disposed of—dismissed. If not at once, but a few days are required to treat and test the largest majority of cases. It is quite impossible to explain satisfactorily a process compassing so many delicate and particular questions within the reasonable limits of a paper. There is a word of caution, I wish to impress with emphasis as to the importance of adjusting the rubber dam, so there will be no possibility of leakage. A very ugly cendition would follow carelessness. Percolations of carbolic acid under the gums would utterly destroy the tissue as far as absorbed; therefore, make no mistake. If the ligature or thread will not accomplish the object, assistance can be obtained from cotton saturated with sandarac varnish—oxy-phosphate can be used also to seal the leak, if carefully manipulated. A precautionary course under all circumstances is to thoroughly absorb the excess on blotting paper, applying the spunk simply moist. There are many methods and systems of practice, all for the accomplishment of the same end with more or less varying success. The interest prevailing at this time, more perhaps than ever heretofore, in efforts to preserve the natural teeth in a normal and useful condition, is highly praiseworthy and can but result in the greatest good, and will award to the successful, the highest attainment in the profession. Our processes may be regarded as heroic, admitting, they are successful. Therefore, we indorse them after a careful test for over a year, and offer them to the profession for their careful consideration.

Mentioning the fact that he who indorses in this rapidly advancing age, only what meets the ready acceptance of his reason and understanding will find himself always in the rear. There are stranger things in heaven and earth Horatio, etc. So, prove all things and hold fast that which is good.

